# A new heterotropic vascularized model of total urinary bladder transplantation in a rat model

**DOI:** 10.1038/s41598-021-83128-w

**Published:** 2021-02-12

**Authors:** Arkadiusz Jundziłł, Henryk Witmanowski, Ewa Żary-Sikorska, Jan Adamowicz, Magdalena Bodnar, Andrzej Marszałek, Tomasz Kloskowski, Kaja Męcińska-Jundziłł, Maciej Gagat, Natalia Siedlecka, Tomasz Drewa, Marta Pokrywczyńska

**Affiliations:** 1 Chair of Urology and Andrology, Department of Regenerative Medicine, Cell and Tissue Bank, Collegium Medicum in Bydgoszcz, Nicolaus Copernicus University in Torun, Skłodowskiej-Curie 9, 85-094 Bydgoszcz, Poland; 2 Department of Plastic, Reconstructive and Aesthetic Surgery, Collegium Medicum in Bydgoszcz, Nicolaus Copernicus University in Torun, Bydgoszcz, Poland; 3 Department of Microbiology and Food Technology, Faculty of Agriculture and Biotechnology, University of Science and Technology, Bydgoszcz, Poland; 4 Department of Clinical Pathomorphology, Collegium Medicum in Bydgoszcz, Nicolaus Copernicus University in Torun, Bydgoszcz, Poland; 5 Department of Clinical Pathology, Poznan University of Medical Sciences and Greater Poland Cancer Center, Poznan, Poland; 6 Chair of Dermatology, Sexually Transmitted Diseases and Immunodermatology, Collegium Medicum in Bydgoszcz, Nicolaus Copernicus University in Torun, Bydgoszcz, Poland; 7 Department of Embryology and Histology Collegium Medicum in Bydgoszcz, Nicolaus Copernicus University in Torun, Bydgoszcz, Poland

**Keywords:** Urinary tract, Bladder

## Abstract

This study developed a new procedure of urinary bladder transplantation on a rat model (n = 40). Heterotopic urinary bladder transplantation (n = 10) in the right groin vessels was performed. Direct urinary bladder examination, microangiography, histological analysis, and India ink injection were performed to evaluate the proposed method's functionality. Observation time was four weeks. One week after the procedure, the graft survival rate was 80%, two urinary bladders were lost due to anastomosis failure. The rest of the grafts survived two weeks without any complications. Lack of transitional epithelium or smooth muscle layer loss and lack of inflammatory process development were observed. This study was performed in order to obtain the necessary knowledge about urinary bladder transplantation. The proposed technique offers a new approach to the existing orthotropic models.

## Introduction

Radical cystectomy is the standard gold method for the treatment of muscle-invasive urinary bladder cancer^1^⁠. Segments of the gastrointestinal tract are utilized for both continent and incontinent urinary diversion, but the last few decades have not been marked by significant technical improvement in this field^2^. The multiple side effects of urinary diversion due to urine interactions with intestinal mucosa trigger new urinary diversion methods following radical cystectomy^3,4^. Besides, after abdominal surgery or radiotherapy, the bowel segment could not be utilized in numerous patients. The numerous successful face transplantations that undertaken their physiological function convinced us to follow up on a successful bladder transplantation procedure. Each year, new, more effective techniques of composite tissue allotransplantation (CTA) are developed, which, together with new immunosuppression methods, can accelerate progress in urinary bladder transplantation^5^. Despite intensive work on the skin composite tissue allograft (CTA) transplantation model development, the idea of human bladder allotransplantation has abounded. The lack of progress in the clinical use of a transplanted bladder is the lack of preclinical studies showing the procedure's feasibility. Experiments on animal models are required to overcome technical and immunological problems to use urinary bladder grafting in clinical practice. The lack of appropriate animal models and small diameters of bladder blood vessels makes the proposed method very demanding with a high probability of complications such as anastomotic and thrombotic complications^6,7^. These technical problems cause that orthotopic urinary bladder transplantation to be characterized by a large number of failures^6^. In order to resolve these difficulties, we developed a new vascular anastomosis model for urinary bladder transplantation. The proposed in this study method can contribute to successful urinary bladder transplantation from cadavers. This procedure may find application in patients in whom standard gastrointestinal urinary diversion is not possible.

## Results

### Anatomical analysis of urinary bladder vascularity

Evaluation of bladders 14 days after right vessels dissection revealed a complete viable urinary bladder wall. Partial bladder atrophy (66%) was noticed in a group with left urinary bladder vessels dissection after 14 days (supplied by adequate, ligated left vascular pedicle). Obtained results showed repeatable anatomic conditions in all exanimated animals. Based on the obtained data, we proved that the whole bladder wall is supplied enough by left bladder vessels. The right bladder vessels are responsible only for 30% of total bladder blood supply. Based on our observations, in the bladder transplantation model, we used the left vascular pedicle.

### Direct bladder evaluation post-transplantation

The four-week follow-up period revealed eight viable grafts and two with lifeless supplying vascular pedicles (n = 2). It was associated with technical failure during microvascular anastomosis.

### Microangiography

Microangiograpic evaluation showed in the 1st Group after right vascular pedicle ligation and in the 3rd Group after urinary bladder transplantation on the left vascular pedicle, full preserved gross vascularization after 2 weeks post-surgical procedure. However, in the 2nd Group with left iliac vessel ligation, the main vessels were noticeable, the size and branches from the right iliac internal artery were poorly developed.

### India ink injection study

**Figure 1:**
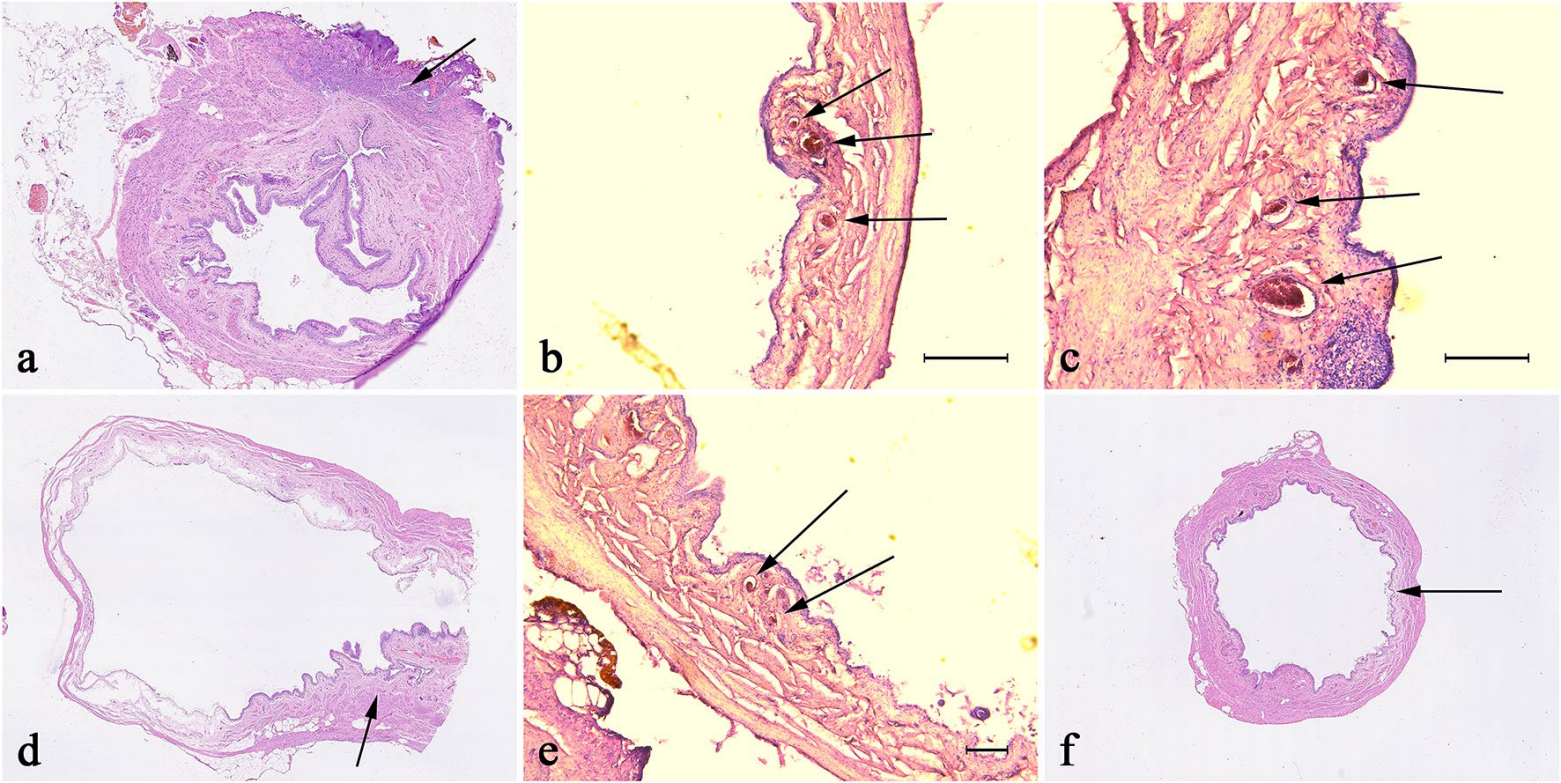
Histological evaluation of India ink injection study. In pictures from (**a**–**c**), we have visible inside vessels contrast marked with arrows. The arrows in the d and e photo indicate the extravasated, inter-tissue contrast after left pedicle ligation (LP). Marked with arrow region in picture correspond with lack of inter-tissue contrast in Group after right pedicle ligation (RP).

Histological analysis indicated India ink dye's presence in the urinary bladder's small-diameter vessels after ligation of the right iliac internal artery and confirmed vascular pedicle patency. The bladder samples from the left ligation iliac internal artery—2nd Group indicated the vascularization border—India ink inherence in small diameter arteries—beginning near right trigonum top encircled near the 30% of the bladder doom (Fig. 1).

### Histologic examination

#### Inflammation

**Figure 2:**
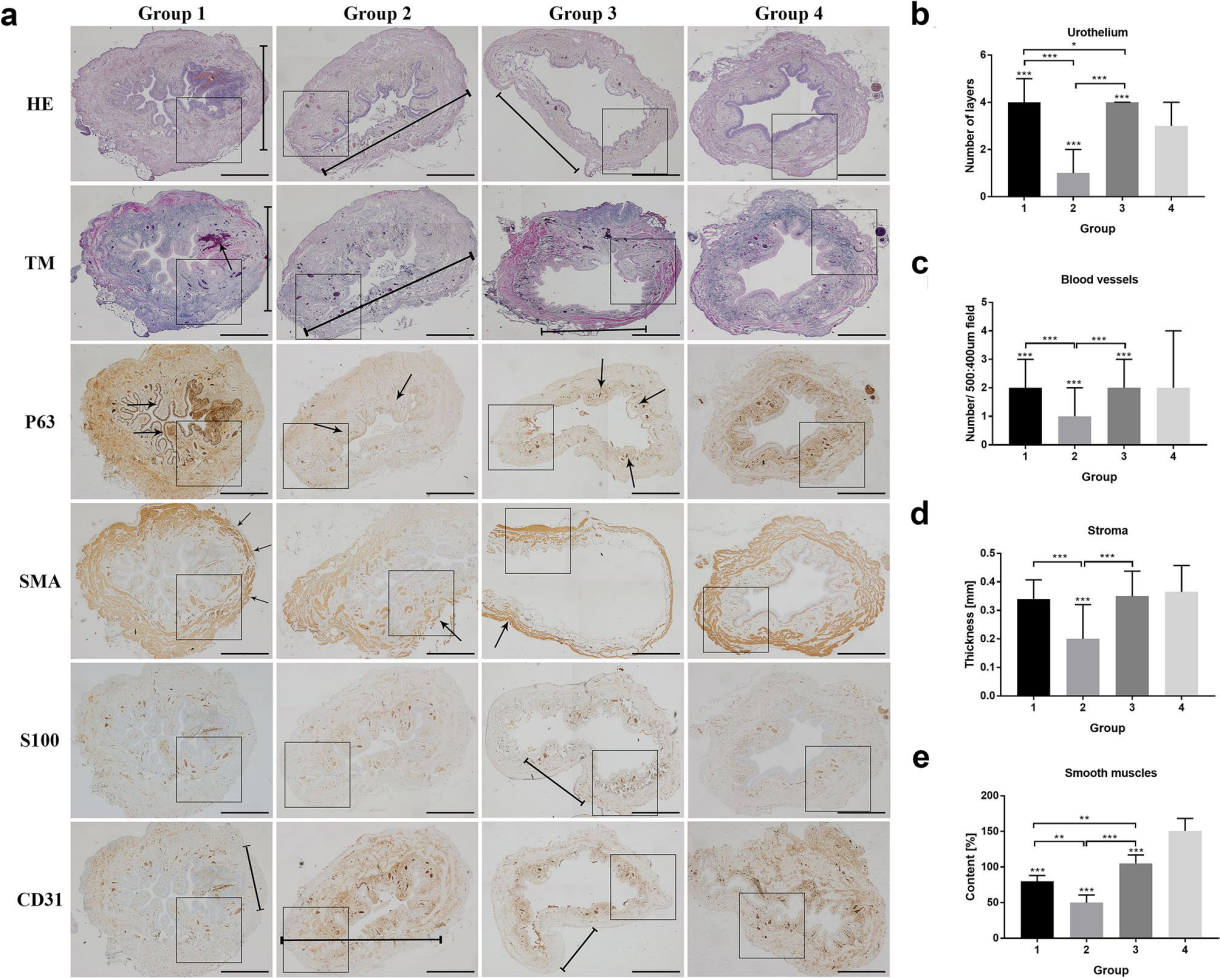
Histological appearance of rat's urinary bladders in each Group with different staining. (**a**) The HE and TM staining in the ischemic wall region indicated in Group 1 and 2 and after reperfusion in Group 3 (marked with a line). The wall ischemia corresponded with the extravasation of Group 1 and the inflammation process in Group 1 (arrow in TM staining), and destruction of bladder wall in Group 3 (marked with a line). Moreover, the P63 staining indicates the hypertrophy of urothelium in a region on a border of ischemia in a Group 1 and 2 and slight hypertrophy in bladder after transplantation due to warm ischemia time (arrows). The SMA staining revealed the variation in muscle arrangement and muscle wall thickness corresponding to the regions of poor blood supply (arrows). The S100 evaluation assessment confirmed fewer nerve fibers in regions with the low blood supply in Group 1, 2, and 3 (marked with a line) and with no differences in Group 4. The CD31 staining confirmed that blood vessels' appearance in a whole bladder wall exempts region without blood supply (marked with a line). Histopathological evaluation of (**b**) number of urothelial layers, (**c**) vessel density, (**d**) bladder wall thickness (**e**) smooth muscle content in each Group, respectively. Bars show the median (**b**–**d**) or mean (**e**); error bars show the interquartile range (**b**–**d**) or SD (**e**). Statistical significance marked with: * < 0.05*, *p < 0.01, ***p < 0. 001. The squares correspond to the enlarged spot in the grid of the higher histopathological evaluation net (Fig. 3.).

**Figure 3:**
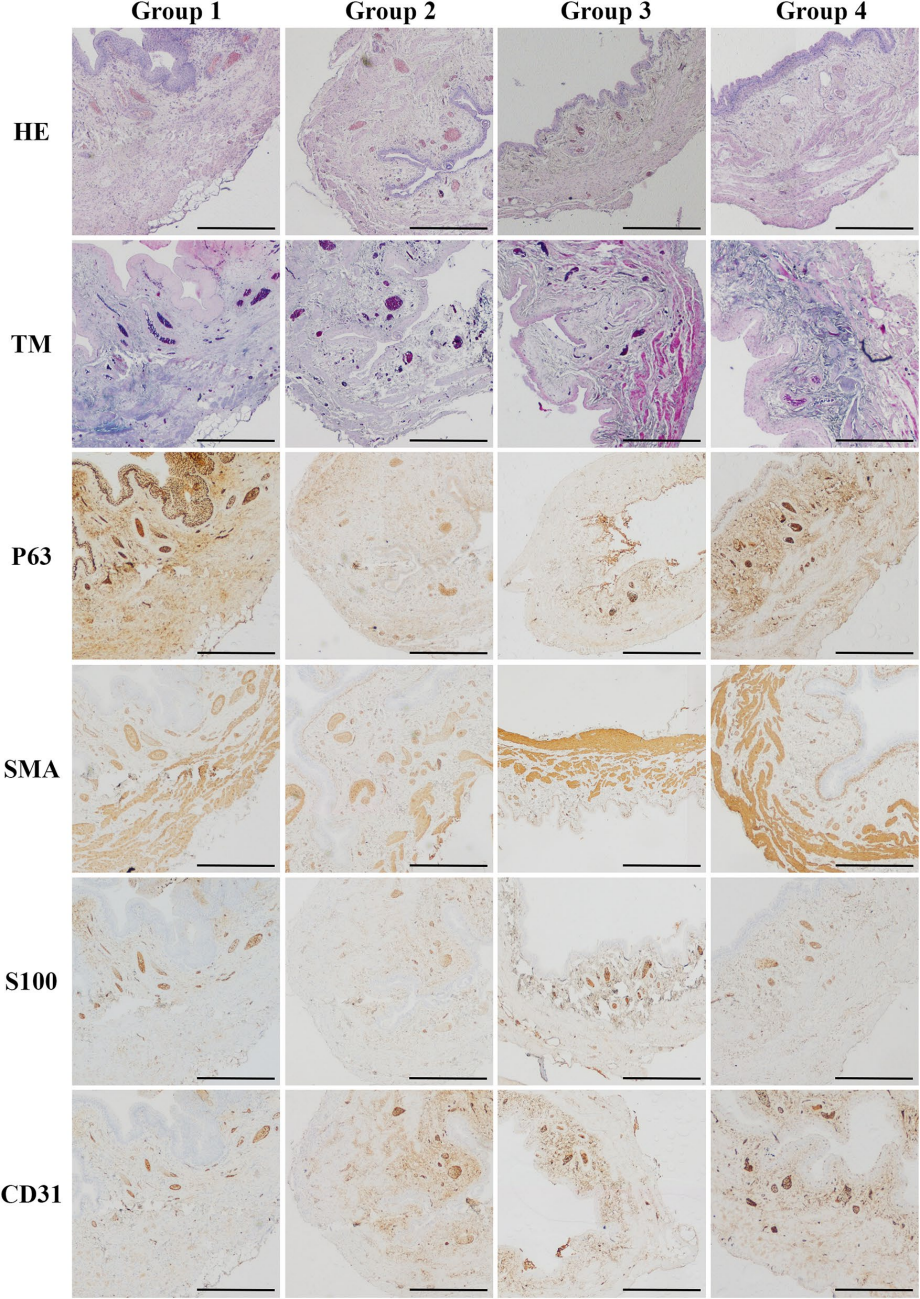
Higher magnification of histological appearance of rat's urinary bladders in each Group with different immunostaining.

The inflammation, edema, and intravascular coagulation were present in Group 1 and more intense in Group 2; however, in Groups 3 and 4, this reaction was mild or absent (Figs. 2 and 3).

#### Urothelium layers

Microscopic evaluation of urothelium layers number revealed the higher median number in Group 1 and 3 and lower in Group 2 compared to control (Group 4). In Group 2, we assessed significant urothelium damage comparing to Group 1 and 3. We even noticed more significant differences in urothelium thickness in Group 1 than in Group 3, which indicates the environmental influence on the bladder transplanted region. The urothelium was slightly hypertrophic in random zones in Group 3, which was confirmed statistically (Figs. 2 and 3). The most irregular arrangement of urothelium layers was present in Group 1; however, in Group 2, this effect was almost invisible (Figs. 2 and 3).

#### Digital evaluation of smooth muscles area

Staining with HE and Masson trichrome showed a decrease in thickness and area of smooth muscle layers in all groups compared to control (Figs. 2 and 3). The differences between the tested groups were observed. The lowest percentage of smooth muscles was observed in Group 2 (48%). In contrast, the number of smooth muscles in a transplanted Group 3 was higher compared to the Group with right pedicle ligation—Group 1 (77% vs. 102). Moreover, the quality of smooth muscle layers in Group 3 was most similar to the control one through all operated groups (Figs. 2 and 3).

#### Stroma thickness

In Group 1, the bladders' wall layers were thinner around 20% in a right coronal plane, supplied by right bladder vessels with no statistical differences than Group 3 and 4. After two weeks, 70% wall atrophy with focal necrotic regions in a group with left pedicle vascular supply ligation was observed. These results confirmed the median evaluation with gross downregulation compared with Group 1, 3, and 4 (*p* < 0.001). The bladder wall's disturbing architecture accompanied extensive infiltration of polymorphic cell inflammation with vascular clots. Histologic examination of the urinary bladder walls revealed complete thickness layers in the 3rd Group after urinary bladder transplantation intro groin area similar to the native one. No significant manifestation of urinary bladder transplant shrinkage or calcification was observed. It is worth noting that the transplanted bladders-maintained organization and architecture despite long ischemia time. However, in the groups where the vessel's blood supply (part of the anatomic study) were performed the differences in the layers' organization were noticeable (Figs. 2 and 3).

#### Blood vessels

The highest number of vessels were observed in the 1st (after right iliac internal artery ligation) and 3rd (transplantation) groups in opposite to low results gained in the 2nd Group (left pedicle transaction) confirmed with significant statistical evaluation. Moreover, histological assessment in Groups 1 and 3 demonstrated no statistical differences in both groups. The number of blood vessels in all operated groups was *p* < 0.001 despite a similar median value. It is worth noting that the sizeable interquartile range slightly affects this results' statistical significance. The histopathological assessment indicated that the ischemic region (created as a result of ligation of the vascular pedicle) impacts the formation of new blood vessels that further organize into the branched microvascular network (Figs. 2 and 3).

## Discussion

The concept of full urinary bladder transplantations is new hope for patients following radical cystectomy. Urinary bladder diversion utilizing the intestinal segment was associated with the long—term, severe complications^11^. Many various techniques have been introduced, ranging from ureterocystoplasty ending on tissue-engineered materials seeded with different cells^12–14^. The proposed in this study technique has many potential benefits. First of all, it is an excellent laboratory model eliminating severe, long-lasting procedures to the recipient in opposite to orthotropic urinary bladder transplantation. A second potential advantage is less technically demanding procedures not involving the uretro- and urethero-vesical anastomoses, which increases transplantation success. The recipient's urinary tract is not involved in a surgical procedure that guarantees the best survival rate. The operator can quickly inspect the transplanted urinary bladder without laparotomy. Even when the vascular pedicle or graft rejection failure appeared, we can easily excise the urinary bladder without losing the recipient essential in transplantation rejection protocols.

Separate urinary bladder transplantation, without the prostate, seminal vesicles or other adjacent organs offer better bladder immunology characterization and better for future immunosuppression development^7,15^.

The inconsistency of insufficient immunosuppression protocols for urinary bladder allotransplantation requires further investigation^16^. Many protocols performed partial urinary bladder transplantation without the trigonum, which constitutes the most critical part of a bladder together with urethral sphincters^13^. Our model includes full bladder transplantation. On the other hand, the lack of graft functionality is the main obstacle to use this model in clinical applications. However, by analogy looking at the routine procedure of iliac kidney transplantation, the urinary bladder transplantation model using iliac vessels without opening the abdominal cavity is a promising alternative in the perspective of bladder transplantation^7^.

Our study is opposite to Yamataka et al., which claimed that neovascularization was established in the urinary bladder after transplantation into peritoneal omentum faster than conventional vascular anastomosis and the time for vesical mucosa regeneration was 3 days compared to 3 weeks respectively^6^. Short cold ischemic time (50 min) in this protocol constitutes fast mucosa regeneration. We confirmed only transient partial bladder mucosa desquamation with simultaneous repopulation. Despite this fact, the thick bladder wall in a large animal model is the main problem for the spontaneous vascularization process^17,18^. In our opinion, despite lifelong immunosuppression side effects, full wall thickness transplantation warrants complete functionality^19^.

## Materials and methods

### Animals

The Nicolaus Copernicus University Ethics Committee approved the protocol (no. 27/2013). According to the Ethical Committee of official European Union recommendations and guidelines (2010/63/EU) of animal use, all experiments were performed. This study was carried out in compliance with the ARRIVE guidelines. Animals were caged at room temperature on a 12-h light/dark cycle. Standard laboratory food and water were available ad libitum. Animals were housed in a barrier animal facility. Operations were performed under anesthesia cocktail of ketamine (30 mg/kg), xylazine (6 mg/kg) and acepromazine (1 mg/kg). Additional doses were given if necessary. Various pain systemic analgesic techniques reduced postoperative pain (combined: non-steroidal anti-inflammatory drugs).

### Urinary bladder vascularity: an anatomic protocol

**Figure 4:**
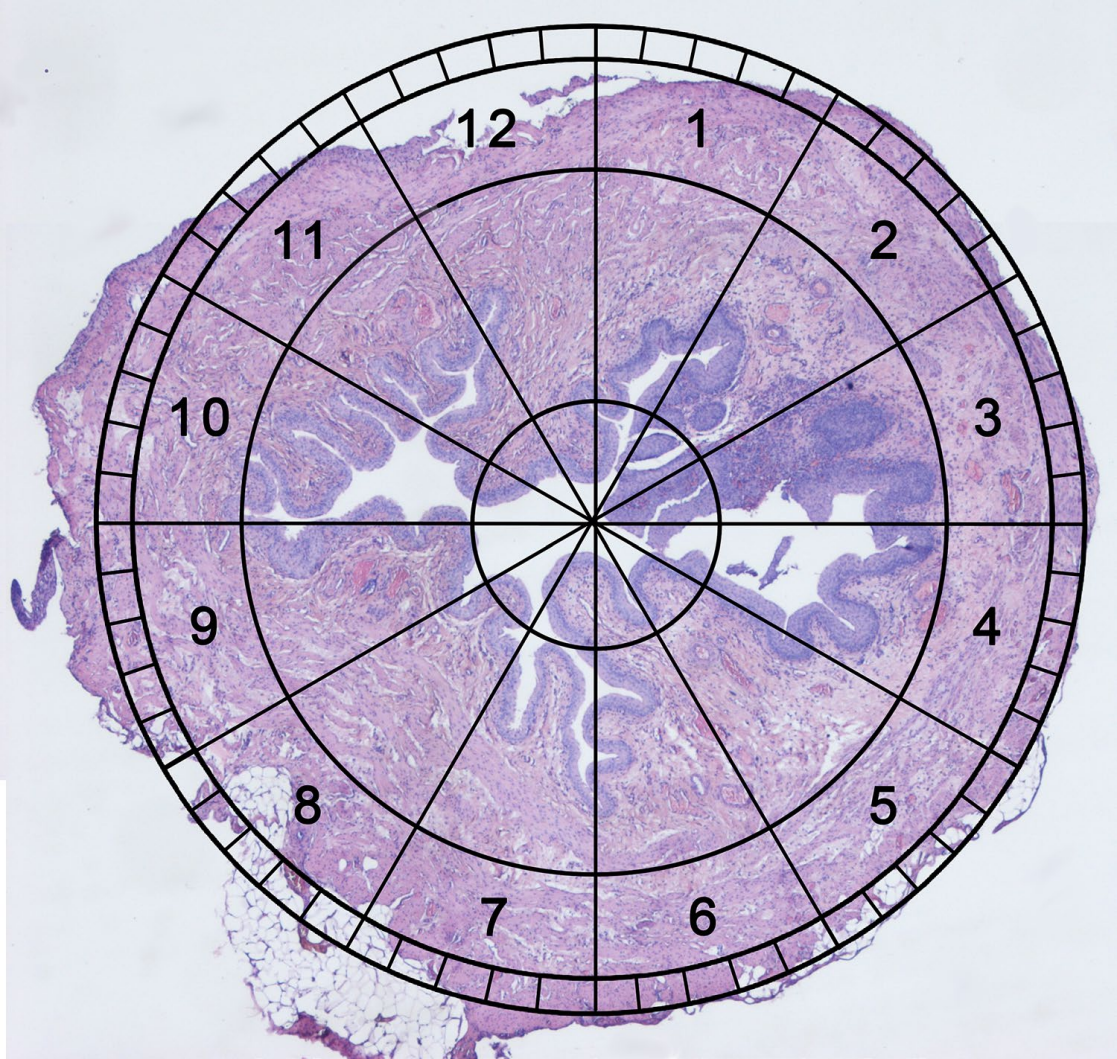
Novel clock system evaluation. An invented, simple method for transverse bladder histological samples assessment using the clock face system with 7 and 6 on the front and 12 and 1 at the back.

Twenty female rats were assessed in this protocol. Animals were used when left internal iliac arteries and veins sufficiently supply the urinary bladder. The abdominal cavity was opened through a midline, lower incision and urinary bladder were exposed using two saline-wetted cotton-tipped applicators. Then both bladder vascular supplies from the internal iliac arteries were localized and marked with a vascular suspender. The same procedure was implemented for marking the ureters. Multiple vascular plexuses between the uterus, bladder, and urethra were cauterized and ligated by titanium clamps to reduce an operation time. Then in the 1st Group (n = 10), the right urinary bladder vessels were ligated and cut. The contrariwise procedure involving left bladder vessels and the right vascular pedicle was performed in the 2nd Group (n = 10). The observation time was two weeks (Fig. 4).

### Urinary bladder transplantation procedure

The donor urinary bladders were obtained from 10 male Wistar rats weighing 300-350 g. Harvested urinary bladders were transplanted into the left groin area of 10 syngeneic female Wistar rats weighing 300- 350 g (3rd group). It was a two-staged operating procedure, including urinary bladder isograft preparation and transplantation. The observation time was four weeks. The control group (4th group) consisted of 10 animals was not involved in any surgical or pharmacological protocols.

### Urinary bladder isograft preparation

After the anesthesia and before the surgery, the abdomen region was trimmed and swabbed with an antiseptic solution. The donor rat was fixed in the supine position. Using lower incision moving intestines (to the left) and rectum (to the right), the urinary bladder and uterus were exposed. Before the main procedure started, two ureters and main urinary bladder arteries and veins were localized. The urinary bladder was carefully prepared not to interrupt the vascular supply from the internal iliac vessels. Multiple vascular plexuses between the uterus and urinary bladder were cauterized. In order to reduce the operation time, the ligations by titanium clamps were used. Following the propagation of the left urinary bladder vessels supply, we prepared them to iliac bifurcation, cutting all vascular branches. Then the urethra and ureters were dissected out from the surrounding tissues as far as possible, ligated, and cut. The uterus and rectum were also ligated and cut. After 1000 U heparinization, left iliac artery and vein double successive clamping, the urinary bladder was excised "en block" with the trigonal region.

### Urinary bladder transplantation

**Figure 5:**
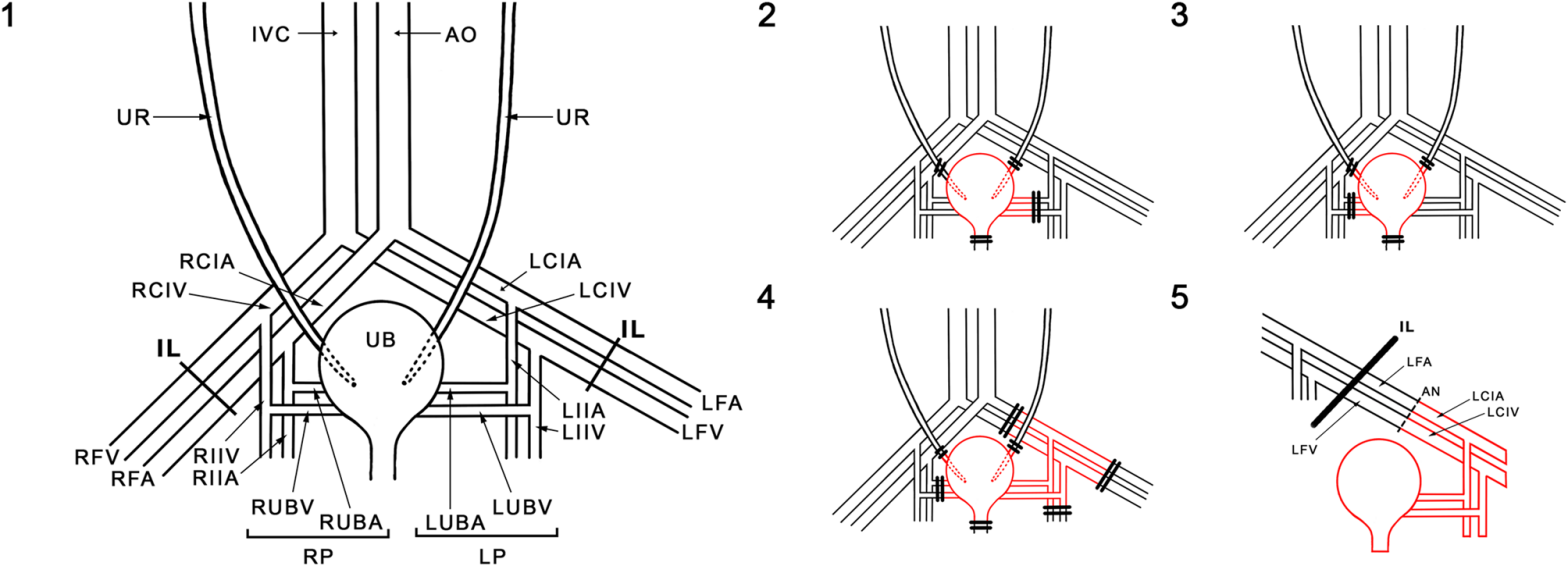
Schematic presentation of bladder vasculature assessment (1–3) and bladder transplantation (1, 4, and 5). *UR* ureter, *IVC* inferior vena cava, *AO* aorta, *RCIA* right common iliac artery, *RCIV* right common iliac vein, *RVA* right femoral artery, *RVF* right femoral vein, *RIIA* right internal iliac artery, *RIIV* right internal iliac artery vein, *RUBA* right urinary bladder artery, *RUBV* right urinary bladder vein; RP (RUBA + RUBV), *UB* urinary bladder, *LUBA* left urinary bladder artery, *LUBV* left urinary bladder vein, LP(LUBA + LUBV), *LIIA* left internal iliac artery, *LIIV* left internal iliac vein, *LFA* left femoral artery, *LFV* light femoral vein, *LIIA* left internal iliac artery, *LIIV* left internal iliac vein, *IL* inguinal ligamentum, *AN* anastomosis.

**Figure 6:**
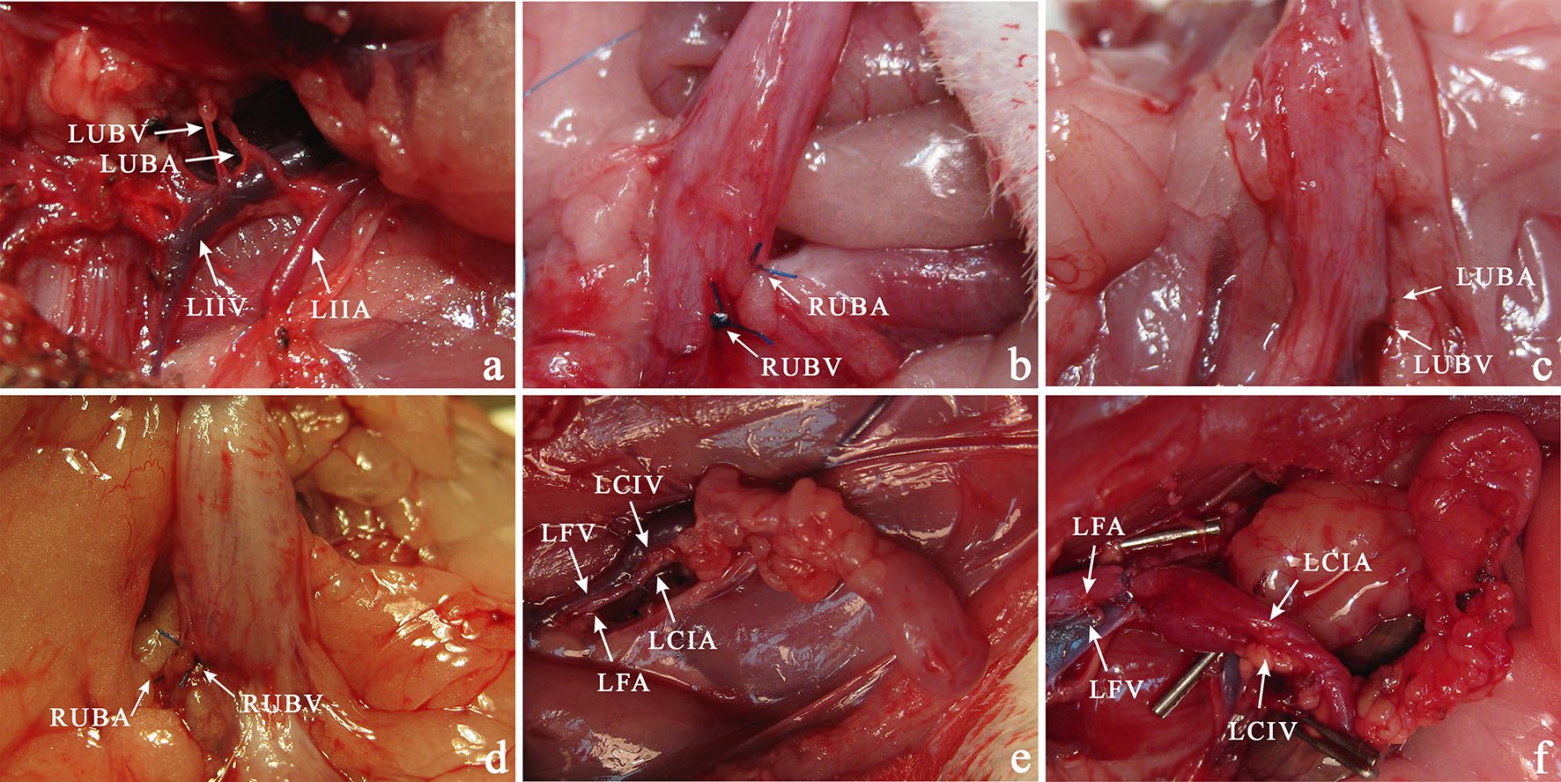
Urinary Bladder vascularity. Anatomic study of the origin of urinary bladder vascular pedicle (**a**). Ligation of the right urinary bladder vascular pedicle (**b**). In two weeks after ligation of the left (**c**) and right (**d**) vascular pedicle, the urinary bladder. Transplantation of the urinary bladder in the left groin area (**e**). Urinary bladder transplanted into the left groin area in one month after surgery (**f**). *LIIA* left internal iliac artery, *LIIV* left internal iliac vein, *LFA* left femoral artery, *LFV* left femoral vein, *LUBA* left urinary bladder artery, *LUBV* left urinary bladder vein, *LCIV* left common iliac vein, *LCIA* left common iliac artery.

In the recipient rat, following incision of the left groin skin, the femoral vessels were exposed and dissected proximally up to the inguinal ligament and distally down to the branching of the superficial epigastric vessels, then ligated and cut. The vascular branches running from and to the femoral artery and vein were ligated and divided. The donor's left vascular urinary bladder pedicle was anastomosed with the recipient's femoral artery and vein stumps using the standard end-to-end interrupted (artery) or continuous (vein) 9.0 nonabsorbable nylon (Ethicon, USA) suture technique (Figs. 5 and 6). We used Cyclosporine A (CsA) protocol in dosage 16 mg/kg/day per os during four weeks observation time. The dosage of CsA was established previously in the CTA skin model. The efficacy of immunosuppression drug administration was randomly monitored by serum level of CsA.

### Direct urinary bladder examination for signs and grade of failure

Urinary bladder graphs were evaluated during two weeks postoperative follow up by gross symptoms of vascular pedicle failure-swelling, redness, abscess in a left groin skin area. The viability of urinary bladder complex graphics was followed by seven days of periodic invasive (open) urinary bladder examination—3 times points evaluations at seven days intervals.

### India ink injection study

After longitudinal abdomen dissection in heterotopic urinary bladder transplant, all the adjacent branches were cauterized. Following cannulation of the left common iliac artery with a 22-gage catheter, 2 ml of India ink was injected. The graft was harvested for hematoxylin and eosin (HE) staining procedure and examination under light microscopy for the presence of India ink in blood vessels of the cross-section through a full urinary bladder wall. Moreover, the India ink injection dye was utilized to assess the left and right pedicle vascularization border in the anatomic examination (1st and 2nd group) at the end of observation lasting two weeks.

### Histologic examination

**Figure 7:**
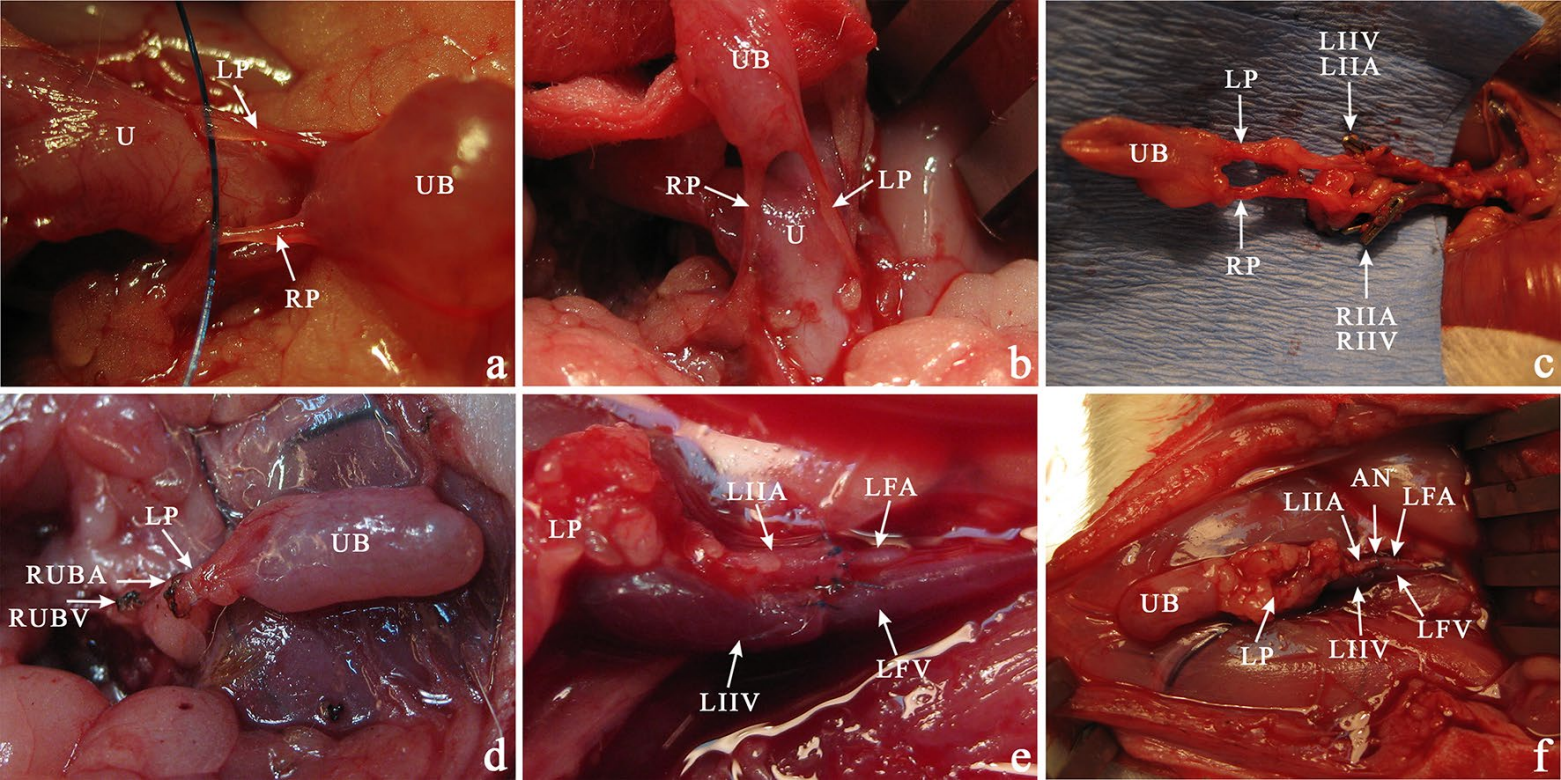
The bladder transplantation procedure. (**a**) The vascular pedicles dissected from the uterus with all adjacent vascular plexuses cauterized hanged on a thread. (**b**) For better visualization of the vascular bundles, the bladder is raised up. (**c**) Following the propagation of the left and right urinary bladder vessels, we dissected the vascular pedicle iliac bifurcation cutting all vascular branches using cauther and titan clamps (arrows). (**d**) The right vascular pedicle was clamped, and after the left iliac artery and vein double successive clamping, the urinary bladder was excised "en block" with the trigonal region. The cauterized vessels are marked with arrows. (**e**) After the femoral artery and vein were exposed, the donors left the vascular urinary bladder pedicle based on the iliac artery, and the vein stump was anastomosed end-to-end interrupted (artery) or continuous (vein) microsurgery technique. (**f**) Successful bladder transplantation in a groin area after 60 min post-transplantation. The vascular pedicle was patent, and all bladders were well blood supplied. Vascular anastomosis marked with an arrow. *UB* urinary bladder, *U* uterus, *LUBA* left urinary bladder artery, *LUBV* left urinary bladder vein, *RUBA* right urinary bladder artery, *RUBV* right urinary bladder vein, LP (LUBA + LUBV), RP (RUBA + RUBV). *LIIA* left internal iliac artery, *LIIV* left internal iliac vein, *LFA* left femoral artery, *LFV* left femoral vein.

Cross-sections of the whole urinary bladder with adjacent tissue and blood supply were fitted in 33 animals. Histological analysis of HE staining was performed. Urinary bladder samples were evaluated for histological symptoms of inflammation, smooth muscle abundance, urothelium hypo or hyperplasia, and stromal thickness reference to 12 radiating zones (numbered 1 to 12 in clockwise order) in a transverse plane. The clock system was placed around the longitudinal axis with 7 and 6 on the front and 12 and 1 at the back (Figs. 5 and 7). Additionally, samples were stained with Masson's trichrome for muscle tissue assessment. The urothelial cell layers were estimated at 40 × light microscope magnification, three assessments per 1 zone. The number of urinary bladder vessels and nerve fiber areas was calculated in 3 different, random areas per one zone located concentrically (500:400 µm each) at 40 × magnification. Image J software was used for morphometric analysis^8^.

### Immunohistochemistry

The immunohistochemical staining was performed according to previously described procedures^9,10^. Tissue sections were incubated with primary (ready to use) antibodies: mouse monoclonal anti-CD31 (cat. No.: IR610), mouse monoclonal anti-p63 (cat. No.: IR662); rabbit polyclonal anti-S100 (cat. No: IR504), mouse monoclonal anti-SMA (cat. No.: IR611) (Dako, Agilent Technologies). The sections were counterstained with hematoxylin, dehydrated in increasing grades of ethyl alcohol, cleared in xylenes, and mounted.

### Microangiography

Angiography was performed in a 1st and 2nd Group to assess bladder vascular pedicle two weeks post-operation procedure. After cannulation of the left common iliac artery with a 22 G catheter, the Uropolinum was injected, and the real-time angiography was performed. The vascular pedicle's patency was assessed by intravenous microangiography using X-ray Actube Dental 5D2 with exposures at 60 kV and six mA.

### Statistical evaluation

The Shapiro–Wilk test was used to assess the normality assumption. Normally distributed (parametric) data were assessed using the one-way Analysis of Variance (ANOVA) followed by the Least Significant Difference (LSD) post hoc test. Non-normally distributed (non-parametric) data were assessed using the Kruskal Wallis test followed by the Mann–Whitney U test with Bonferroni correction.

## Data Availability

All data generated or analyzed during this study are included in this published article.
